# Home care challenges and adaptive capacity in patients with left ventricular assist devices: an evidence-informed qualitative study

**DOI:** 10.1186/s13019-026-04568-x

**Published:** 2026-07-15

**Authors:** Jiahong Tian, Ling xia, Jingyu Hua, Liping Xuan, Li Fang, Cheng Chen, Yingying Sang

**Affiliations:** 1 Department of Nursing, Anhui Chest Hospital, Hefei, 230000 China; 2 Cardiovascular Surgery, Anhui Chest Hospital, Hefei, 230000 China

**Keywords:** Left ventricular assist device, Care needs, Descriptive qualitative interview, Evidence-informed

## Abstract

**Objective:**

To identify post-discharge home care challenges and adaptive capacities among patients with left ventricular assist devices (LVADs), and to inform strategies for optimizing transitional care and long-term heart failure management in the Chinese healthcare context.

**Methods:**

A two-phase, evidence-informed qualitative design was employed. In phase one, a structured literature review of six databases was conducted, and eight relevant studies were synthesized to inform the development of a semi-structured interview guide. In phase two, in-depth telephone interviews were conducted with 13 patients during home-based rehabilitation following LVAD implantation. Interview data were analyzed using reflexive thematic analysis.

**Results:**

Based on the interview data, 52 sub-codes were identified and organized into 29 care need themes across four domains: self-management, medical care, device management, and social support. Key needs included emotional and family support, standardized device and wound care, emergency preparedness, rehabilitation guidance, medical accessibility, battery management, and financial or caregiving resource support. Participants also demonstrated situated adaptive capacity in managing device-related and home-care demands.

**Conclusion:**

Patients with LVADs face substantial post-discharge home management challenges, while vulnerability and adaptive capacity coexist during recovery. These findings support a context-sensitive, patient-centered transitional care approach that strengthens self-management, caregiver involvement, device adaptation, and continuity of care in heart failure pathways.

**Supplementary Information:**

The online version contains supplementary material available at https://doi.org/10.1186/s13019-026-04568-x.

## Introduction

First introduced in the early 1960s and termed a “bridge to recovery,” the left ventricular assist device (LVAD) has brought hope to patients with end-stage heart failure by offering a vital treatment option for those awaiting heart transplantation or deemed ineligible for transplantion [1, 2]. With the launch of the first fully magnetically levitated LVAD in China in 2021, this technology has become increasingly available to patients with advanced heart failure, marking an important step in the development of artificial heart therapy within China’s heart failure surgical management system [3]. However, LVAD implantation does not signify the end of a patient’s recovery journey. Instead, the long-term reliance on the device introduces complex, multidimensional challenges. These issues increasingly underscore the urgent need for systematic identification and response to patients’ ongoing care needs.

Although numerous studies have explored post-LVAD care experiences, the majority adopt a phenomenological methodology, often lacking effective integration between theoretical frameworks and clinical practice. Grounded in an evidence-based foundation, the present study conducted a systematic literature review to extract key issues, which were then used to inform a descriptive qualitative interview. Accordingly, this study aimed to explore the post-discharge home care challenges, care needs, and adaptive capacity of patients living with LVADs, and to generate evidence-informed insights for improving transitional care and long-term heart failure management.

## Participants and methods

### Participants and setting

Using purposive sampling, patients were recruited from the Department of Cardiovascular Surgery at a hospital in China. Eligible participants were those who had undergone left ventricular assist device (LVAD) implantation and were receiving home-based rehabilitation between December 2025 and March 2026. Inclusion criteria were as follows: (1) patients in postoperative recovery at home, and (2) ability to engage in verbal communication. Exclusion criteria included: (1) presence of psychiatric disorders, (2) severe comorbid organic diseases, and (3) critically ill status at the time of recruitment.

Ethical approval was obtained from the institutional Ethics Committee of Anhui Chest Hospital, China (Approval No. 2025-001-01). The consent process and data collection procedures were conducted in accordance with the approved ethical protocol. The semi-structured interview guide was developed based on the results of a prior systematic review of the literature. Sample size was determined to ensure coverage of all major dimensions of care needs and was guided by the data adequacy, in which sampling continued until no new meaningful patterns were identified [4]. A total of 13 participants were ultimately included in the study.

### Research methods

This study adopted a descriptive qualitative approach following the framework of Braun and Clarke’s reflexive thematic analysis [5, 6], and was conducted in two sequential phases that incorporated both an evidence-informed literature review and qualitative interviews. The aim was to develop an evidence-informed interview framework and explore patients’ home care challenges and adaptive experiences through qualitative interviews. In this design, the literature review was used to inform the interview guide and contextualize the discussion, whereas the qualitative findings reported in the Results were generated from participants’ interview data.A detailed flowchart outlining the study process is presented in Fig. 1.

**Fig. 1:**
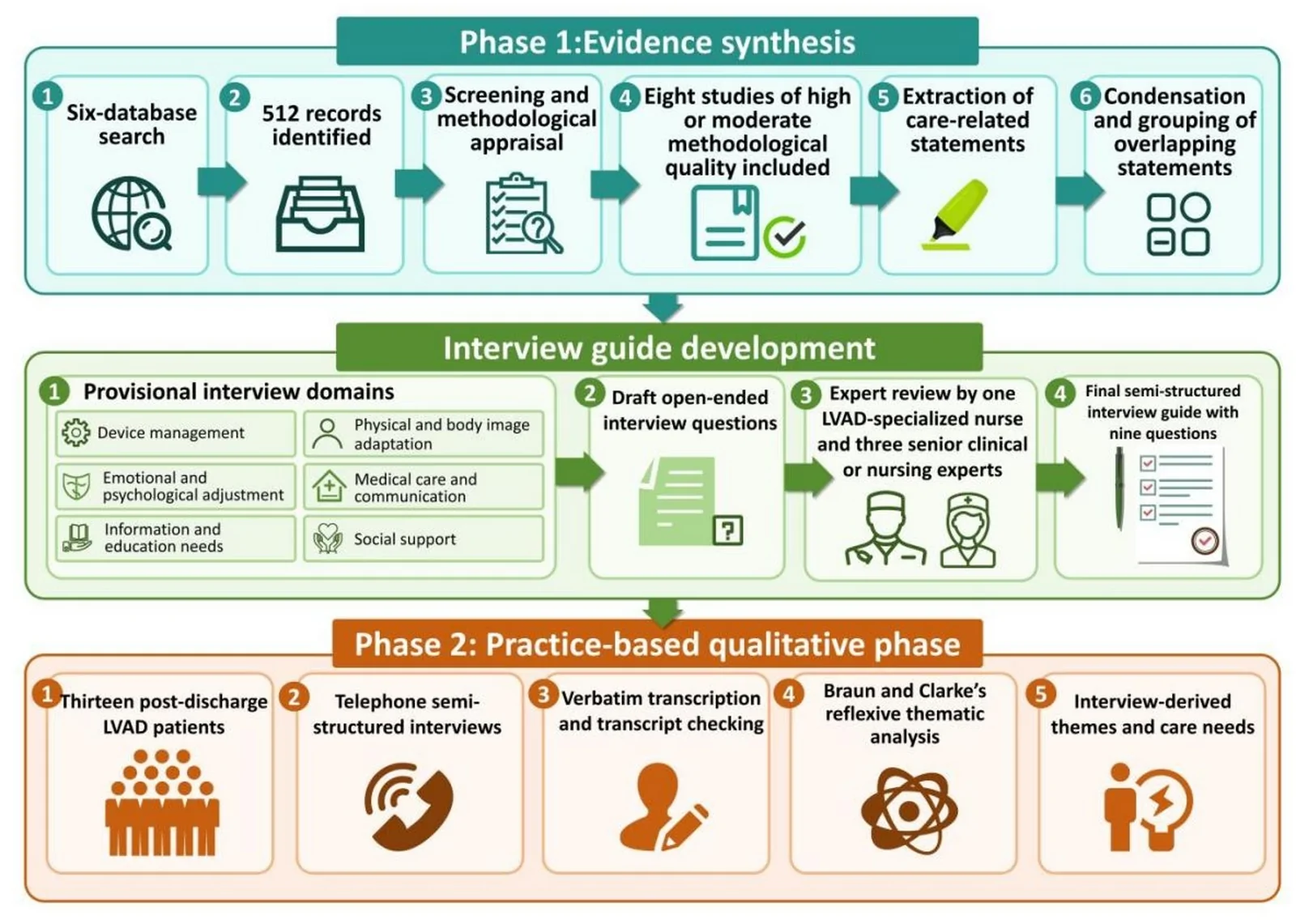
Evidence-informed development of the interview guide and qualitative analysis process

#### Evidence synthesis phase

① **Literature search and screening** For Chinese-language databases (SinoMed, CNKI, and Wanfang), equivalent Chinese search terms were applied, corresponding to the following concepts: “left ventricular assist device,” “care needs,” “subjective experience,” “qualitative study,” “systematic review,” and “evidence-based nursing.” A total of 512 articles were initially identified. Inclusion criteria for study selection were: (1) studies involving patients who had undergone LVAD implantation or their caregivers; (2) content addressing care needs or rehabilitation experiences; and (3) study type categorized as a systematic review, qualitative evidence synthesis, primary qualitative study, meta-analysis, or relevant quantitative study addressing LVAD-related device use, patient experience, or home management. Following screening and quality assessment, eight studies of high or moderate methodological quality were included. We included systematic reviews, qualitative evidence syntheses, primary qualitative studies, and relevant quantitative studies if they addressed LVAD-related home care, patient experience, device use, self-management, or support needs.

The methodological quality of the included studies was appraised using tools selected according to study design. The CASP Qualitative Checklist was used for primary qualitative studies, whereas the appropriate JBI critical appraisal checklist was used for systematic reviews, qualitative evidence syntheses, meta-analyses, and quantitative or mixed-design studies. To improve transparency, the appraisal tool used for each included study has been added to Table 1. Five studies were rated as high quality and three as moderate quality. Key characteristics of the included studies are summarized in Table 1. The extracted content from these studies is detailed in Appendix 1.

② **Development of the interview guide and quality control** Based on the content of the eight included studies, care-related statements relevant to post-LVAD home management were extracted and preliminarily organized into interview domains. First, two researchers independently reviewed the eight included studies and extracted care-related statements. In this study, care-related statements were defined as descriptions of post-discharge difficulties, daily management tasks, device-related concerns, self-care behaviors, rehabilitation experiences, emotional responses, information needs, family or professional support, and social or financial barriers relevant to living with an LVAD at home.

**Table 1:** Basic characteristics of included studies

No.	First Author (year)	Study type	Sample size/evidence base	Appraisal tool used	Main care-related content extracted	Quality rating	Evidence contribution to interview guide
1	Dunn, J.L. (2019) [7]	Systematic Review	35 studies	JBI critical appraisal tools	Equipment burden and emotional response	High	Device management, alarm response, wearability
2	Friedman, J.A. (2020) [8]	Systematic Review + Qualitative Synthesis	6 studies	JBI checklist for systematic reviews / evidence syntheses	Perception of dying and emotional regulation	High	Emotional adjustment, preoperative information needs
3	Kitko et al.(2016) [9]	Primary qualitative study	15 participants	CASP Qualitative Checklist	Information and social support needs	High	Information needs, social support
4	Li, Wanhui et al. (2023) [10]	Qualitative meta-synthesis	15 studies	JBI checklist for systematic reviews / evidence syntheses	Benefit from rehabilitation exercises and adapt to challenges	High	Rehabilitation, physical adaptation, coping
5	Schloglhofer et al.(2023) [11]	Cross-sectional quantitative study	58 participants	JBI checklist according to study design	Operational and behavioral limitations	Moderate	Device use, battery management, mobility restrictions
6	You N, et al. (2024) [12]	Qualitative meta-synthesis	10 studies	JBI checklist for systematic reviews / evidence syntheses	Decision-making difficulties and risk perception	High	Risk perception, preoperative information needs
7	Li F et al. (2024) [13]	Primary qualitative study	10 participants	CASP Qualitative Checklist	Multidimensional caregiving support needs	Moderate	Medical care, family caregiving, home support
8	Zhai ZC, et al. (2025) [14]	Primary qualitative study	13 participants	CASP Qualitative Checklist	Psychological and caregiving continuity challenges	Moderate	Home rehabilitation, psychological support, continuity of care

**Table 2:** Interview guide for post-LVAD patients

No.	Dimension	Interview question	Supporting study No. in Table 1
1	Device Management Needs	When using the device at home, what worries or concerns you the most? Is there any part that you find particularly difficult to operate?	1,2,5
2	Device Management Needs	What do you usually do when the device gives an alarm? How did you feel the first time you heard it?	1,5
3	Physical and Body Image Adaptation	Since having the device implanted, what changes have you noticed in your body? Were there any unexpected changes?	4,6
4	Physical and Body Image Adaptation	While living at home, does the device ever interfere with your ability to dress, shower, or move around? Are there any parts you find inconvenient?	1,5
5	Emotional and Psychological Adjustment	During recovery, have there been times when you felt stressed, tired, or emotionally overwhelmed? How did you handle those moments?	2,4,6
6	Social Support Needs	Are there moments when you especially wished to talk to someone or hoped someone would visit you?	3,4
7	Medical Care Needs	After discharge, do you feel it’s easy to get in touch with your doctor or nurse when needed? If there’s an issue with the device, who do you rely on?	3,7
8	Information and Education Needs	Thinking back to before your surgery, is there anything you wish the doctor or nurse had told you in advance—something like, “I wish I had known that”?	3,6
9	Information and Education Needs	If someone who knows little about this device were to ask you about it, what would you most want to tell them? Or what would you want a doctor/nurse to explain for you?	2,6,8

Second, the extracted statements were compared, condensed, and grouped through discussion. Similar or overlapping content was merged, while statements reflecting different aspects of home-based adaptation were retained. This process generated several provisional interview domains, including device management, physical and body image adaptation, emotional and psychological adjustment, medical care and communication, information and education needs, and social support.

Third, these provisional domains were translated into open-ended interview questions. The questions were designed to elicit patients’ lived experiences and practical concerns rather than to confirm pre-existing categories from the literature. Each question was linked to the corresponding evidence source in Table 1 to ensure that the interview guide was grounded in prior evidence while remaining open to new findings from participants’ narratives.

Finally, the draft interview guide was reviewed by one LVAD-specialized nurse and three senior clinical or nursing experts. All experts were from Anhui Chest Hospital. They were asked to comment on the completeness, relevance, logical sequence, terminology, and patient-centered wording of the interview questions. Their written feedback was summarized by the research team, and revisions were made through team discussion to improve the clarity and clinical relevance of the interview guide. The finalized interview guide is presented in Table 2.

#### Practice-based phase: validation of care needs

① Due to the nature of telephone interviews, written consent was not feasible. Verbal informed consent was obtained prior to each interview. While the verbal consent process was not audio-recorded or transcribed in full, participants’ agreement was acknowledged and documented by the research team through contemporaneous field notes, in accordance with the approved ethical protocol.

② **Qualitative interviews** In-depth semi-structured telephone interviews were conducted using a one-on-one, open-ended format. Each session lasted approximately 15 to 30 min and was audio-recorded. Transcription was completed within three days of each interview, producing verbatim transcripts. To ensure accuracy, the interviewer and a research team member performed a one-on-one cross-check of the transcribed content. The general characteristics of the interview participants are summarized in Table 3.

**Table 3:** General characteristics of interview participants (*n* = 13)

No.	Sex	Age (years)	Education level	Preoperative EF(%)	Weeks post-Op	Weeks since discharge home from index hospitalization	No. of medications	No. of comorbidities	LVAD product/model
P1	Male	53	No formal education	27	24	21	11	11	CorHeart 6
P2	Male	56	Junior high school	34	9	6	5	9	CorHeart 6
P3	Male	54	Primary school	25	14	12	7	9	EVAHEART 2
P4	Male	55	Associate degree	30	6	3	10	12	EVAHEART 2
P5	Male	58	Primary school	27	17	14	7	7	EVAHEART 2
P6	Male	68	Primary school	37	11	7	11	7	EVAHEART 2
P7	Female	67	High school	37	6	2	9	14	EVAHEART 2
P8	Male	42	Bachelor’s degree	21	17	14	10	5	EVAHEART 2
P9	Male	72	High school	34	11	7	7	8	EVAHEART 2
P10	Female	43	Junior high school	26	5	2	10	3	EVAHEART 2
P11	Male	38	Junior high school	25	24	21	10	13	EVAHEART 2
P12	Male	62	Junior high school	22	18	14	7	6	CorHeart 6
P13	Male	53	No formal education	23	24	21	11	6	EVAHEART 2

“No formal education” indicates that the participant had no formal schooling

2. “Weeks since discharge home from index hospitalization” refers to the interval between discharge home from the index hospitalization for LVAD implantation and the interview. None of the participants were transferred to a separate post-acute inpatient rehabilitation unit or rehabilitation facility before returning home

3. LVAD product/model was extracted from the participants’ medical records. Device information was included because differences in external components, battery systems, device weight, alarm settings, and wearability may influence patients’ home care needs and adaptation experiences

③ **Coding analysis and theme development** Verbatim transcripts were imported into NVivo 20.0 for data management and analysis. Data were analyzed following Braun and Clarke’s reflexive thematic analysis framework [5, 6]. Two researchers independently read the transcripts several times, wrote preliminary analytic notes, and generated initial codes that remained close to participants’ original expressions [7].

The coding and theme development process involved three iterative steps. First, the two researchers independently coded the transcripts and identified codes related to daily care tasks, device-related concerns, emotional responses, family support, medical communication, and adaptation strategies. Second, they compared codes, discussed overlapping or divergent interpretations, and returned to the original transcripts and illustrative quotations when disagreements occurred. Third, related codes were grouped into candidate themes and subthemes, which were reviewed against both coded extracts and the full dataset to ensure coherence, distinctiveness, and relevance to the study aim. Coding notes, theme development records, and discussion summaries were retained as an audit trail. The coding and theme development process is summarized in Supplementary Figure S1.

Although the interview guide was informed by prior evidence, the literature-derived domains were not used as a fixed coding framework. During analysis, the researchers remained open to meanings and concerns not anticipated in the interview guide. Team discussions were used to examine whether candidate themes were grounded in participants’ accounts rather than imposed by prior literature or clinical assumptions.

④ **Reflexivity**: The research team included members with backgrounds in cardiovascular surgery and nursing, and some members had experience in LVAD-related care, discharge education, or follow-up management. This background helped the team understand device-related issues, but it may also have sensitized the researchers to clinical risk, device management, and continuity-of-care concerns. To address this influence, the team used reflexive notes, repeated review of transcripts, and discussion of alternative interpretations. Recruitment and analysis continued until no new meaningful patterns relevant to the research aim were identified.

## Results

The findings reported in this section are based on the qualitative interview data rather than on the literature review. A total of 52 sub-codes were identified, reflecting participants’ post-discharge home care challenges, care needs, and adaptive experiences following LVAD implantation. These sub-codes were further synthesized into 29 detailed care need themes across four domains: self-management, medical care, device management, and social support. The complete list of sub-codes, care need themes, coding logic, and reporting decision trail is provided in Appendix 2.

For reporting clarity, Table 4 presents 14 analytically salient care needs that illustrate the main patterns within the four domains. These care needs were not selected through quantitative counting of references. Instead, reporting decisions were made through reflexive discussion within the research team, based on relevance to the study aim, clarity and richness of supporting quotations, relevance to post-discharge safety, self-management, device adaptation, continuity of care, or social reintegration, and representation across the four domains. In the self-management domain, participants described needs related to emotional adjustment, family support, adaptation to the LVAD device, and day-to-day self-care. In the medical care domain, care needs focused on patient-centered health education, caregiver-assisted device care, remote communication, rehabilitation support, and access to home-based medical services. In the device management domain, participants emphasized emergency preparedness for device malfunction, emotional coping during alarms, and battery management for safe travel. In the social support domain, participants described needs related to life reconstruction, reduced work capacity, financial burden, and access to caregiving supplies.

**Table 4:** Fourteen analytically salient home care needs following LVAD implantation

Care domain	Care goal	Care need	Illustrative quote
**Self-management Needs**	Support phased emotional recovery and psychological adaptation	Provide staged rehabilitation feedback and systematic emotional support interventions	**P3**: “After the surgery, it’s not like before when I was constantly out of breath. Now I can talk and walk without feeling exhausted.” **P8**: “Well, at least it saved my life. Without this thing, I wouldn’t be here. Sure, it’s not convenient, but I’m alive—and that’s what matters.” **P13**:“I had to have the surgery—if I didn’t, I wouldn’t have made it. Now I can walk, I can eat—it made a real difference.”
	Enhance emotional support from family members	Establish a structured family support mechanism and peer encouragement system	**P1**: “The kids came to visit that day, and I was so happy I skipped my afternoon nap.” **P2**: “I saw another patient—he seemed to be recovering really well. That gave me confidence, and now I feel like I’m doing pretty well too.” **P5**: “It definitely feels better when there’s someone to talk to.”
	Facilitate adaptation to the LVAD device	Alleviate the burden of device use by offering more convenient and user-friendly alternatives	**P2**: “It gets frustrating—I feel like snapping sometimes. Life’s just too inconvenient now.” **P5**: “Can’t they make a smaller one or something?” **P6**: “If I’d known it was this heavy, I wouldn’t have gone through with it. I’d rather just die—it’s way too much trouble.” **P8**: “It just keeps beeping non-stop—‘beep beep beep’—and even hitting the mute button doesn’t help.”
	Optimize postoperative self-care behaviors	Strengthen postoperative self-care guidance, with a focus on diet management, wound care, and showering practices	**P1**: “I’m mostly worried the part where the tube connects to my body might get bent.” **P3**: “I just have rice porridge every day, a steamed bun, and maybe a bit of pickled vegetables.” **P11**: “Before I got sick, I was afraid to eat anything. Now I can eat again—and I eat quite a bit.”
**Medical care needs**	Strengthen self-care capacity and decision-making ability	Deliver patient-centered health education tailored to individual understanding and needs	**P7**: “Oh, I’m not sure about that. I’ll ask my son or husband when they get back. I don’t really understand it—no one ever explained it to me.” **P12**: “The dressing changes have been going fine. The wound isn’t red or swollen.” **P13**: “Yeah, I’ve heard the alarm before—I just call my daughter over when it happens.”
	Standardize home-based device operation practices	Enhance the competencies of family caregivers and standardize home-based dressing changes	**P9**: “I change the dressing twice a day—been doing it all along.” **P11**: “My wife does the dressing changes for me.”
	Establish a multi-channel emergency response system	Maintain accessible telecommunication channels and optimize telehealth services to improve medical accessibility	**P1**: “Whenever we post something in the WeChat group, the doctor or nurse replies right away—it’s really convenient.” **P5**: “If there’s anything I can’t figure out, I go straight to the engineers. Like when the alarm goes off and I don’t know what to do, I ask them to handle it.” **P8**: “When it really comes down to it, it’s the hospital and the doctors who can save me—that’s how I see it.”
	Assess physical capacity and promote rehabilitation exercise	Develop individualized physical assessment and rehabilitation plans	**P4**: “I don’t dare go out—I just feel like I don’t have the strength.” **P9**:“Since I got home, I haven’t done any heavy activity.” **P10**: “I go up and down the stairs every day, and I take walks too.”
	Improve accessibility to medical services	Offer in-home medical visits and provide personalized recommendations based on device type, usage habits, and the actual home environment	**P6**: “It’s pretty easy to get in touch with the medical staff—but the hospital is just too far away.” **P8**: “I keep thinking, if something urgent happens, your hospital is just too far from here.” **P8**: “Could you provide some videos on how to handle emergencies?”
**Device management needs**	Develop emergency management strategies for device malfunction	Enhance training on backup device use and establish emergency protocols for device malfunction	**P7**: “What I’m really afraid of is the device breaking. If it breaks… doesn’t that mean I’d die?” **P12**: “What scares me most right now is the device breaking—and then not having anything to rely on.”
	Support emotional coping during device alarms	Implement emotional support and technical assistance for managing device alarms	**P3**: “As soon as it starts beeping, my heart just races—I get really scared.” **P5**: “Yeah, I do get a little scared when it starts going off.”
	Address battery anxiety and support safe travel	Improve patients’ battery management skills and develop detailed travel preparedness plans	**P5**: “I do feel a bit anxious about the battery running out.” **P10**: “The battery doesn’t last that long, so I always bring two when I go out. If it looks like it’s running low, I switch it right away.”
**Social support needs**	Gradually restore work capacity	Provide life reconstruction support for patients facing reduced work capacity and mobility limitations	**P2**: “I used to be able to work, but now I can’t anymore.” **P4**: “I don’t dare go out—I just feel like I don’t have the strength.” **P12**: “With this machine on me, there are just things I can’t do—not even small tasks.”
	Reduce financial burden and improve access to caregiving supplies	Strengthen economic assistance policies and improve access to essential medical supplies and caregiving resources	**P1**: “I wish insurance could cover more. The price could definitely be lower—otherwise, it’s really hard to afford staying alive.” **P6**: “That waterproof cover is just too expensive.” **P11**: “Even sterile water for injection is hard to get.”

## Discussion

The interview-derived care needs identified in this study showed broad consistency with prior LVAD-related evidence, while also reflecting several context-specific features of local practice. In relation to device-related care, participants described battery-related anxiety, difficulty wearing or carrying the device, and alarm-related disruption, which were consistent with findings reported by Dunn [8] and Schlöglhofer [9] .In relation to self-management, themes such as rehabilitation, emotional adjustment, and family support aligned with previous findings by Friedman, Kitko, and Li et al. [10–12].Medical and social support needs, including continuity of care, emergency preparedness, financial burden, and social acceptance, were also consistent with the work of You et al., Li et al., and Zhai et al. [13–15]. In addition to these consistencies, the present study identified locally relevant issues, including expectations for lighter-weight domestic devices and improved access to home-based medical services. Based on these findings, the following discussion is organized around device-integrated care, mind-body empowerment, whole-process care, implications for heart failure management, and social inclusion.

It is important to interpret these findings within the context of Chinese culture and the Chinese healthcare delivery system. In this setting, family members often play a central role in post-discharge care, including dressing changes, device observation, emotional support, and decision-making during emergencies. At the same time, participants’ reliance on WeChat groups, direct contact with hospital professionals, and device engineers reflected a locally embedded pattern of informal but highly practical communication after discharge. Compared with more established LVAD care pathways in some North American and European settings, structured home-based LVAD nursing services and community-based support systems remain less standardized in the present context. Therefore, the care needs identified in this study should be understood not only as individual patient experiences, but also as responses shaped by family caregiving norms, regional healthcare accessibility, emerging home-care services, and the characteristics of domestically used LVAD systems.

### Device-integrated care: from functional adaptation to living in symbiosis

Left ventricular assist devices (LVADs) have emerged as a critical therapeutic option for patients with end-stage heart failure. Their effectiveness in extending survival and improving postoperative quality of life has been well documented in both domestic and international studies [1, 16–18]. However, the long-term coexistence with the device presents persistent psychological and lifestyle challenges for patients following surgery.

Unlike earlier studies that primarily emphasized survival outcomes, participants in the present study described the LVAD as a “life crutch,” indicating a desire not only to prolong life but also to improve its quality. For instance, participants P1 and P12 reported that the Corheart-6 device, produced by a locally developed device, facilitated recovery due to its lightweight construction and minimally invasive implantation technique. In contrast, the Chongqing Yongrenxin LVAD was perceived by several participants as cumbersome, limiting mobility and overall comfort. Participants expressed strong expectations for the development of lighter, more easily replaceable external components to enable “painless” upgrades, thereby improving postoperative quality of life. Similar design efforts have been observed in devices such as the HeartWare HVAD system developed in the United States [16, 19].

### Mind-body empowerment: striking a dynamic balance between emotional adjustment and family support

Despite the challenges associated with long-term device dependence, most post-LVAD patients experience a process of psychological self-adjustment, gradually adapting to their new way of life [10]. In the present study, participant P7, a female patient, expressed a notable fear of device malfunction, stating, “If it breaks down, wouldn’t that mean I die?” This sentiment is consistent with findings by Blumer, who reported that female patients are more likely to exhibit device-related anxiety in a large-scale quality-of-life study involving 4,493 individuals [20].

Family support has been identified as a critical factor in facilitating recovery and adjustment in home-based settings [21]. Beyond their role as caregivers, family members serve as essential partners in helping patients navigate postoperative adaptation. It is recommended that professional nursing staff provide targeted education and guidance to family members to improve the quality and effectiveness of home support, thereby contributing to patients’ physical and psychological well-being [22].

Furthermore, evidence indicates that structured postoperative rehabilitation can significantly improve ventricular function and reduce the risk of long-term disability [23]. Patients should undergo individualized assessment of their functional capacity and be encouraged to engage in progressive rehabilitation programs. Strengthening home-based rehabilitation guidance may further support recovery and facilitate the resumption of daily activities [24].

### Whole-process care: advancing from skill training to health empowerment

Although definitive evidence regarding the optimal duration of nursing follow-up remains lacking, the importance of continuity of care is well recognized [25]. Patients living with LVADs, in particular, require the development of accessible and responsive communication systems to manage potential complications in a timely manner. At the same time, patients themselves remain their primary caregivers. Therefore, their self-care abilities and clinical decision-making capacity should be carefully assessed and systematically reinforced, regardless of educational background [26].

Additionally, many patients in this study reported improvements in physical status following surgery, suggesting the feasibility of implementing phased rehabilitation assessments during home visits. Providing professional feedback at each stage may help to enhance patients’ emotional motivation and promote active participation in the recovery process.

These findings are consistent with primary studies from North American LVAD populations showing that patients and caregivers continue to encounter self-management difficulties after discharge and require competency assessment, reassessment, and ongoing communication with the VAD care team [27]. However, the present study also shows a context-specific pattern. Rather than describing formalized post-discharge training programs, participants more often referred to practical and accessible support, such as WeChat-based communication, direct contact with clinicians or engineers, video-based emergency teaching, and expectations for home visits. This suggests that in the Chinese context, whole-process care may need to combine standardized LVAD self-management education with flexible communication channels and locally feasible home-based support.

### Implications for heart failure management

These findings extend the relevance of this study from postoperative nursing care to the broader field of advanced heart failure management. In patients supported with LVADs, treatment success should not be defined solely by survival or device performance, but also by the patient’s ability to sustain self-care, maintain functional adaptation, and manage uncertainty after discharge [26].

This interpretation is also supported by recent LVAD self-management science. International studies have increasingly conceptualized LVAD care as a multidimensional self-care process that includes monitoring, heart failure-related self-care, and LVAD-specific self-care [28]. Similarly, intervention studies from North American settings have tested structured approaches such as smartphone app-directed self-management support [29] and simulation-based mastery learning to improve patient and caregiver self-care skills [30]. Compared with these intervention-oriented approaches, the present study highlights an earlier step in the care pathway: identifying which needs are most salient to patients in a Chinese post-discharge context before designing formal interventions. Our findings therefore complement existing LVAD self-management research by showing how device management, heart failure management, family-supported care, and healthcare accessibility intersect in patients’ daily lives.

The findings also suggest that LVAD patients should not be understood only through a vulnerability-oriented or device-dependence lens. Although participants reported fear of device malfunction, alarm-related anxiety, caregiver dependence, limited mobility, and financial pressure, some also demonstrated adaptive agency in daily life. For example, participants described monitoring wound condition, seeking family support during alarms, carrying spare batteries when going out, gradually resuming walking or stair climbing, and reframing the LVAD as a life-sustaining support rather than only as a burden. These findings indicate that adaptive capacity is not a uniform personal trait, but a situated process shaped by the device, family support, professional guidance, and the home environment. Recognizing this capacity may help clinicians design care that protects patients while also mobilizing their existing self-management potential.

### Social inclusion: a coordinated pathway toward resource integration and life reconstruction

The presence of an LVAD often results in changes to patients’ physical appearance and behavioral patterns, which may pose challenges to social recognition and acceptance. International studies have projected a growing population of LVAD recipients, highlighting the need for greater visibility and acknowledgment of their social and economic contributions [31].

Improving public awareness of LVAD-related health conditions may contribute to enhanced societal acceptance and reduced stigma associated with device dependence. At the policy level, there is a need to strengthen the integration of healthcare services with community-based support, thereby establishing diversified systems that facilitate the reintegration of LVAD patients into daily social life.

### Limitations

This study has several limitations. First, participants were recruited from a single hospital in China, and the sample size was limited to 13 patients. Although the sample was sufficient for identifying meaningful patterns within this qualitative study, the findings may not fully represent the experiences of LVAD patients in other regions, institutions, or healthcare systems. Second, data were collected through telephone interviews. This approach improved accessibility for patients during home-based rehabilitation, but it limited the researchers’ ability to observe non-verbal expressions and contextual details in the home environment. Third, the study focused on patients’ post-discharge experiences at one period of home-based recovery; therefore, it may not capture changes in care needs and adaptive capacity over longer periods. Finally, this study included only patient perspectives. Future research should consider multi-center and longitudinal designs, and include caregivers, nurses, physicians, and device engineers to develop and evaluate more comprehensive transitional care interventions.

## Conclusion

Based on an evidence-informed interview framework, this study identified 14 core home care needs among patients following LVAD implantation, offering insight into their lived experiences, adaptive capacities, and care expectations.

Unlike traditional qualitative studies that emphasize detailed intervention proposals, the present study prioritizes directional thinking in nursing care design. It highlights the importance of context-specific strategies that address essential care needs, rather than applying generalized templates that may lack empirical validation.

In recent years, qualitative research has increasingly focused on mapping comprehensive patient needs. However, care needs should not be interpreted only through a vulnerability-oriented lens. This study suggests that vulnerability and adaptive capacity coexist in the home-based recovery of LVAD patients. Some participants developed practical strategies for device management, family-supported coping, gradual activity recovery, and uncertainty management after discharge. Centering care around core needs may therefore help clinicians move beyond an overprotective view of LVAD patients and design strategies that both safeguard patients and strengthen their existing self-management potential.

Future research should explore the development and validation of targeted interventions based on core needs and examine their effectiveness in enhancing care quality and continuity across broader clinical contexts.

## Supplementary Information

Supplementary Material 1

Supplementary Material 2

## Data Availability

The data that support the findings of this study are available from the corresponding author upon reasonable request. Due to the nature of qualitative data and ethical restrictions, full transcripts are not publicly available.
