# Supplementary material for: Home care challenges and adaptive capacity in patients with left ventricular assist devices: an evidence-informed qualitative study
Source: J Cardiothorac Surg. 2026 Jul 15;21:752. doi: 10.1186/s13019-026-04568-x (PMC13651043; doi:10.1186/s13019-026-04568-x)
Supplement: Supplementary file 1 [file 13019_2026_4568_MOESM1_ESM.docx]

**Appendix 2. Theme development and reporting decision trail**

Appendix 2 presents the coding logic and reporting decision trail from sub-codes to care needs. “Reference point, n” refers to the number of coded extracts related to each sub-code and is provided only to show the recurrence of participants’ concerns within the dataset. It was not used as a quantitative criterion for determining theme importance. Reporting decisions were made through reflexive discussion within the research team, based on the relevance of each sub-code or theme to the study aim, the clarity and richness of supporting quotations, and its relevance to post-discharge safety, self-management, device adaptation, continuity of care, or social reintegration. Care needs reported in Table 4 were selected because they were analytically salient and representative of the four overarching domains, rather than because they had the highest reference count.

| **Dimension** | **Theme** | **Current Situation Description** | **Reference Point, n** | **Care Need** | **Coding logic and reporting rationale** | **Reporting status in Table 4** |
| --- | --- | --- | --- | --- | --- | --- |
| **Self-management Needs** | Emotional and Psychological Adaptation | A01 Improved postoperative health enhances rehabilitation confidence | 26 | Positive feedback; no further needs identified. Reinforce recovery progress to enhance positive perception. | Significant rehabilitation improvement observed; positive changes should be communicated systematically. | Directly reported in Table 4 |
|  |  | A02 Actively adjusting mindset and self-perception of functional recovery | 17 |  |  | Merged under Table 4 item |
|  |  | A03 Psychological comfort provided by family | 11 | Strengthen family and peer support systems.  Reduce device burden; advocate for manufacturers to produce compact, compatible components for replacement. | Strong emotional dependence; social interaction enhances motivation for recovery.  Device negatively affects quality of life; clear emotional resistance is present. | Directly reported in Table 4 |
|  |  | A05 Need for social companionship | 8 |  |  | Merged under Table 4 item |
|  |  | A08 Encouragement from others' successful recovery experiences | 3 |  |  | Directly reported in Table 4 |
|  |  | A04 Device weight limits mobility and causes psychological distress | 8 | Develop individualized physical rehabilitation plans, alleviate postoperative fatigue, and offer emotional counseling pre- and post-operation.  Positive feedback only; no further needs converted. | Phased negative emotions emerge; proactive psychological intervention is needed.  Self-care awareness exists, but behaviors vary in quality and standardization; risk of overeating identified. | Directly reported in Table 4 |
|  |  | A06 Perceived device burden offsets rehabilitation benefits | 7 |  |  | Merged under Table 4 item |
|  |  | A07 Device interferes with daily activities | 7 |  |  | Directly reported in Table 4 |
|  |  | A13 Device weight and size restrict mobility | 26 |  |  | Directly reported in Table 4 |
|  |  | A10 Emotional low during early recovery stage | 2 | Enhance postoperative self-care guidance, especially on common behaviors such as dietary management. | Driveline affects dressing convenience; lifestyle adjustment support is needed. | Merged under Table 4 item |
|  |  | A11 Negative preoperative attitude toward surgery | 1 |  |  | Merged under Table 4 item |
|  |  | A12 Improved health leads to better sleep quality | 1 | Provide staged rehabilitation feedback and systematic emotional support interventions |  | Contextual/adaptive finding |
|  |  | A14 Awareness and actions related to postoperative self-care | 14 | Provide dressing and clothing adaptation guidance to reduce driveline interference. | Foreign body sensation impairs body image and requires emotional and psychological support. | Directly reported in Table 4 |
|  |  | A15 Postoperative dietary concerns and lack of fluid balance management | 13 |  |  | Directly reported in Table 4 |
|  |  | A16 Driveline affects daily clothing choices | 6 | Address body image disturbances caused by the device. | Anxiety over driveline safety undermines psychological security and confidence in action. | Merged under Table 4 item |
|  |  | A17 Sensation of physical abnormality due to implanted device | 2 | Facilitate adaptation to the LVAD device | This sub-code reflected patients’ perception of bodily change and foreign-body sensation after implantation. | Merged under Table 4 item |
|  |  | A18 Worry about driveline loosening or damage affecting safety | 1 | Enhance driveline protection and safety education. | Sternal pain indicates varied recovery progress; pain location and intensity should be monitored. | Directly reported in Table 4 |
|  |  | A19 Slow sternum recovery with pain during activity | 1 | Develop individualized physical assessment and rehabilitation plans |  | Merged under Table 4 item |
|  | Anxiety and Risk Perception | A20 Avoidance of showers to prevent wound infection | 18 | Monitor and reassess postoperative pain points continuously. | Bathing avoidance due to perceived infection risks compromises quality of life. | Directly reported in Table 4 |
|  |  | A21 Fear of pain or improper technique during dressing change | 1 | Enhance the competencies of family caregivers and standardize home-based dressing changes |  | Merged under Table 4 item |
| **Medical Care Needs** | Continuity of Care Needs | A22 High dependence on caregiver | 15 | Provide specific guidance on shower timing and protection techniques after surgery.  Strengthen dressing change training procedures. | Fear of pain and mistakes causes tension during dressing changes; confidence and skills need enhancement.  Discrepancies observed between patient and caregiver practices in wound care. | Merged under Table 4 item |
|  |  | A25 Wound care performed by family members | 6 |  |  | Directly reported in Table 4 |
|  |  | A26 Ability to regularly change dressings and assess wound condition | 6 |  |  | Directly reported in Table 4 |
|  |  | A23 Convenience of using WeChat and other informal communication channels | 10 | While improving caregiver skills and standardizing home wound care, patients should also be prepared to manage emergencies.  Maintain accessible telecommunication channels and optimize telehealth services | Physician-patient communication lacks consistency and standardization. | Directly reported in Table 4 |
|  |  | A27 Contacting engineers first in case of device malfunction | 4 |  |  | Directly reported in Table 4 |
|  |  | A31 Prioritizing trust in healthcare professionals during critical moments | 2 |  |  | Directly reported in Table 4 |
|  |  | A09 Postoperative physical decline | 3 | Maintain convenient remote communication channels and optimize responsiveness of postoperative telehealth services.  Design personalized exercise rehabilitation programs. | Recovery of physical capacity varies widely; fear and lack of systematic guidance are common.  Provides insights for optimizing in-home medical visit services. | Directly reported in Table 4 |
|  |  | A24 Difficulty in regaining normal physical activity and lack of motivation | 7 |  |  | Directly reported in Table 4 |
|  |  | A33 Young patients willing to recover but lack systematic guidance | 1 |  |  | Merged under Table 4 item |
|  |  | A28 Long travel distances for follow-up visits | 3 | Provide in-home visits and environment modification suggestions tailored to device characteristics, usage habits, and actual living conditions. | Some patients attempt dressing changes at community clinics. | Directly reported in Table 4 |
|  |  | A29 Home environment modifications to accommodate the device | 3 |  |  | Merged under Table 4 item |
|  |  | A30 Some patients choose community doctors for dressing changes | 2 | Improve coordination with primary care systems / standardize home-based care |  | Merged under Table 4 item |
|  |  | A32 Decreased contact with doctors as physical condition normalizes | 1 | Improve coordination with primary care systems. | Patient preference for self-management suggests follow-up protocols should allow flexibility. | Merged under Table 4 item |
|  | Information and Educational Needs | A34 Adequate coverage of postoperative education content | 7 | Deliver patient-centered health education tailored to individual understanding and needs |  | Merged under Table 4 item |
|  |  | A35 Lack of preoperative awareness about device weight | 2 | Adjust follow-up frequency based on individual recovery status. | While most patients find the education comprehensive, individualized understanding should still be ensured. | Merged under Table 4 item |
|  |  | A36 Need for video-based teaching in emergencies | 1 | Maintain accessible telecommunication channels and optimize telehealth services |  | Directly reported in Table 4 |
|  |  | A37 Difficulty understanding information provided by engineers | 1 | Continuously optimize educational coverage post-surgery and enhance feedback mechanisms. | “Device weight” becomes a common source of regret, reflecting a gap between preoperative expectations and postoperative reality. | Merged under Table 4 item |
| **Device Management Needs** | Difficulties in Device Operation | A38 Anxiety about device failure or malfunction threatening life | 10 | Enhance training on backup device use and establish emergency protocols |  | Directly reported in Table 4 |
|  |  | A39 Gradual mastery of device operation and maintenance | 9 | Bridge preoperative education gaps regarding real-world device experiences. | Patients expect video-based, reviewable instructions for managing unexpected situations. | Merged under Table 4 item |
|  |  | A40 Noisy device operation at night | 4 | Develop accessible, multimodal educational tools (e.g., videos). | Communication with engineers is often hindered by language or cognitive mismatch. | Merged under Table 4 item |
|  |  | A42 Excessive heat during device operation | 2 |  |  | Merged under Table 4 item |
|  |  | A41 Complex maintenance procedures cause distress | 3 | Enhance training on backup device use and establish emergency protocols |  | Merged under Table 4 item |
|  | Alarm Management | A43 Adapted to alarm prompts and able to respond independently | 11 | Improve language alignment and plain-language communication in postoperative guidance. | Heavy dependence on the device; malfunctions are perceived as life-threatening, increasing psychological burden. | Merged under Table 4 item |
|  |  | A44 Alarms trigger fear and anxiety | 4 |  |  | Directly reported in Table 4 |
|  | Power/Charge-related Anxiety | A45 Anxiety caused by battery power levels | 7 | Establish emergency response plans and backup strategies for device malfunction. | Physical discomforts such as noise and heat disturb sleep and overall comfort. | Directly reported in Table 4 |
| **Social Support Needs** | Social Inclusion | A46 Postoperative mobility status and ability to go out | 7 | Provide life reconstruction support for reduced work capacity and mobility limitations |  | Merged under Table 4 item |
|  |  | A47 Attention drawn by device when going out | 6 |  | Learning barriers and perceived complexity of operation reduce patient confidence in self-management. | Merged under Table 4 item |
|  |  | A48 Outings limited by physical strength and device weight | 5 |  |  | Directly reported in Table 4 |
|  |  | A49 Difficulty returning to work post-surgery | 4 | Optimize sensory experience of device operation (e.g., noise reduction, temperature control). | Despite alarm familiarity, some patients panic during alerts, impairing judgment and response. | Directly reported in Table 4 |
|  | Financial Support | A50 High medical expenses cause financial stress | 4 | Reduce financial burden and improve access to caregiving supplies |  | Directly reported in Table 4 |
|  |  | A51 Cost of replacing accessories and consumables causes financial burden | 4 |  |  | Directly reported in Table 4 |
|  |  | A52 Difficulty obtaining maintenance supplies in daily life | 1 |  |  | Directly reported in Table 4 |

Note:“Reporting status in Table 4” indicates whether a sub-code was directly reported in Table 4, merged under a reported Table 4 care need, or retained as a contextual/adaptive finding that informed interpretation but was not separately reported. Cell shading is used only to improve readability. Reporting status is determined by the written label rather than by color.
