# Supplementary material for: Home care challenges and adaptive capacity in patients with left ventricular assist devices: an evidence-informed qualitative study
Source: J Cardiothorac Surg. 2026 Jul 15;21:752. doi: 10.1186/s13019-026-04568-x (PMC13651043; doi:10.1186/s13019-026-04568-x)
Supplement: Supplementary file 2 [file 13019_2026_4568_MOESM2_ESM.docx]

**Supplementary material：**

**Supplementary Figure S1. Reflexive coding and theme development process**

Note: This figure summarizes the iterative process used to move from verbatim transcripts to data-grounded themes. The analysis involved independent initial coding, code comparison, return to original transcripts and illustrative quotations, reflexive discussion of researcher assumptions, theme development and refinement, and maintenance of an audit trail. Team discussion was used to enhance interpretation rather than to force consensus or impose pre-existing frameworks.
